# Sodium-Glucose Cotransporter 2 (SGLT2) Inhibitors: Guardians against Mitochondrial Dysfunction and Endoplasmic Reticulum Stress in Heart Diseases

**DOI:** 10.1021/acsptsci.4c00240

**Published:** 2024-10-16

**Authors:** Linh Thi Truc Pham, Supachoke Mangmool, Warisara Parichatikanond

**Affiliations:** † Biopharmaceutical Sciences Program, Faculty of Pharmacy, Mahidol University, Bangkok, 10400 Thailand; ‡ Department of Pharmacology, Faculty of Pharmacy, Mahidol University, Bangkok, 10400 Thailand; § Department of Pharmaceutical Care, Faculty of Pharmacy, Chiang Mai University, Chiang Mai, 50200 Thailand

**Keywords:** SGLT2 inhibitors, heart diseases, mitochondrial dysfunction, ER stress

## Abstract

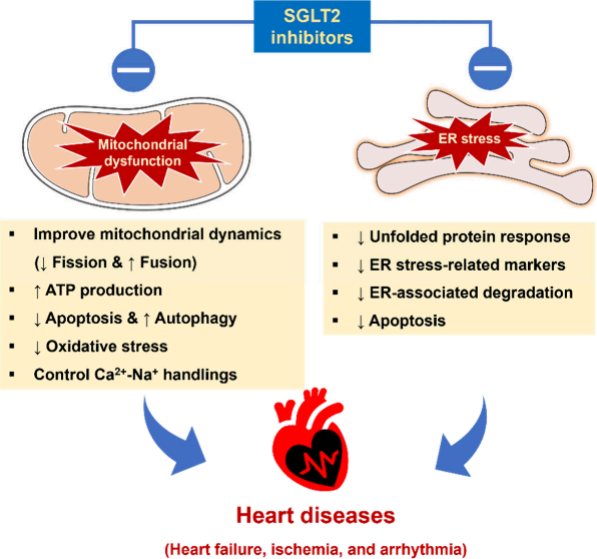

Sodium-glucose cotransporter 2 (SGLT2) inhibitors are an innovative class of antidiabetic drugs that provide cardiovascular benefits to both diabetic and nondiabetic patients, surpassing those of other antidiabetic drugs. Although the roles of mitochondria and endoplasmic reticulum (ER) in cardiovascular research are increasingly recognized as promising therapeutic targets, the exact molecular mechanisms by which SGLT2 inhibitors influence mitochondrial and ER homeostasis in the heart remain incompletely elucidated. This review comprehensively summarizes and discusses the impacts of SGLT2 inhibitors on mitochondrial dysfunction and ER stress in heart diseases including heart failure, ischemic heart disease/myocardial infarction, and arrhythmia from preclinical and clinical studies. Based on the existing evidence, the effects of SGLT2 inhibitors may potentially involve the restoration of mitochondrial biogenesis and alleviation of ER stress. Such consequences are achieved by enhancing adenosine triphosphate (ATP) production, preserving mitochondrial membrane potential, improving the activity of electron transport chain complexes, maintaining mitochondrial dynamics, mitigating oxidative stress and apoptosis, influencing cellular calcium and sodium handling, and targeting the unfolded protein response (UPR) through three signaling pathways including inositol requiring enzyme 1α (IRE1α), protein kinase R like endoplasmic reticulum kinase (PERK), and activating transcription factor 6 (ATF6). Therefore, SGLT2 inhibitors have emerged as a promising target for treating heart diseases due to their potential to improve mitochondrial functions and ER stress.

Mitochondria and the endoplasmic reticulum (ER) are complex intracellular structures essential for cell survival. Mitochondria play major roles in adenosine triphosphate (ATP) production, the synthesis and detoxification of reactive oxygen species (ROS), the upkeep of antioxidant glutathione, the balance of cytoplasmic calcium (Ca^2+^) levels, and the regulation of mitochondrial matrix superoxide dismutase (SOD). Thus, they facilitate the transport of metabolites, influence programmed cell death, and are involved in cell development.^1^ To govern crucial cellular homeostatic functions, mitochondria establish a close connection with the ER with the smooth ER responsible for various metabolic processes and Ca^2+^ storage, while the rough ER is engaged in protein synthesis and post-translational modifications.^2^ Dysfunction in both mitochondria and ER has been associated with a range of cardiovascular diseases (CVDs) including arrhythmias, heart failure (HF), ischemia, hypertrophy, and fibrosis.^3^ Due to the high energy demands, the heart has metabolic flexibility as it can utilize various substrates such as fatty acids, glucose, ketone bodies, and amino acids for ATP replenishment to support its vital functions.^4^ Specifically, cardiac ATP is mainly derived from fatty acid oxidation; however, there is a shift toward increased glucose metabolism during stress conditions.^5^

In humans, cardiac cells express two families of glucose transporters which are facilitated diffusion glucose transporters (GLUTs) and sodium-glucose cotransporters (SGLTs).^6,7^ Particularly, GLUT1 and GLUT4 are the major glucose transporters in the heart and their expressions are regulated by different insults such as chronic hypoxia, long-term fasting, insulin depletion, fatty acids, and thyroid hormone.^6^ In addition to GLUTs, glucose can be transported by the SGLT family with at least SGLT1 and sodium-myoinositol cotransporter-1 (SMIT1) being identified as expressed in the heart.^7^ Remarkably, although only minimal levels of SGLT2 mRNA have been detected in heart tissue and its presence in the heart has not been conclusively proven,^8,9^ numerous preclinical studies have revealed protective effects of SGLT2 inhibitors in isolated cardiomyocytes and hearts, indicating the possibility of off-target actions. Studies using wild-type and SGLT2-deficient mice have confirmed that SGLT2 inhibitor demonstrates cardioprotective effects regardless of SGLT2 presence including reducing infarct size during acute cardiac ischemia/reperfusion (I/R) injury^10^ as well as providing protection against systolic and diastolic dysfunction and myocardial hypertrophy in a murine HF model.^11^

CVD continues to be a major cause of mortality in chronic conditions with the most recent report showing a 48.6% prevalence of CVD in adults of all genders over the 2017–2020 period.^12^ Diabetes is one of the major factors contributing to CVD,^13^ however, adults with diabetes exhibit a variety of CVD risk profiles and diabetes alone confers nonequivalent CVD risk.^14^ Globally, 6.1% of people of all ages have diabetes in 2021 and this number is expected to surpass 10% by 2050.^15^ SGLT2 inhibitors are one of the drug classes that have been approved by the US Food and Drug Administration (US FDA) in the management of type 2 diabetes mellitus (T2DM). Many clinical trials of SGLT2 inhibitors showed positive results of cardiovascular (CV) outcomes in HF patients with diabetes and nondiabetes.^16^ Numerous studies have investigated the role of SGLTs in mitochondrial and ER dysfunction in various tissues including the heart and revealed that inhibiting SGLT2 activity effectively alleviates these abnormalities in both *in vitro* and *in vivo* studies.^17−23^ As a result, SGLT2 inhibitors are emerging as a promising option for the management of heart diseases, although there is no evidence that the cardiac benefits are directly due to SGLT2 inhibition rather than other off-target effects.

## Mitochondrial and ER Function in the Heart

### Function of the Mitochondria in Cardiac Health

Mitochondria possess a double membrane comprising outer and inner layers with the intermembrane space between them and a central matrix.^24^ Within this structure, the inner mitochondrial membrane is folded inward to form cristate which contains enzyme complexes responsible for oxidative phosphorylation. Each mitochondrion is an “ATP factory” which can yield 15 times more ATP than glycolysis alone, with 30 molecules of ATP produced for each molecule of glucose oxidized, and its releasing energy is used as heat to maintain body temperature, a process known as nonshivering thermogenesis.^25,26^ During oxidative phosphorylation, protons are pumped across the inner mitochondrial membrane creating a proton gradient. The majority of these protons flow back into the mitochondrial matrix through ATP synthase generating ATP. However, some protons leak back into the matrix through uncoupling proteins (UCPs), causing the dissipation of energy as heat rather than being used for ATP synthesis.^27,28^

In cells with high energy needs, there is a higher number of observed mitochondria compared to that in cells with a low-energy metabolism. For example, it was estimated that an adult ventricular myocyte contains approximately 7,000 mitochondria and 800 mitochondria per hepatocyte while only a small number of mitochondria was found around the axoneme of the sperm tail with none in red blood cells.^29,30^ Mitochondria are crucial for numerous cellular activities including ATP synthesis, neutralizing intracellular and mitochondrial ROS, maintaining antioxidant glutathione levels, regulating Ca^2+^ levels, facilitating metabolite transport, and participating in programmed cell death and cell development.^1^ Consequently, mitochondrial dysfunction is associated with the pathogenesis of various types of CVDs.

### Contribution of the ER to Heart Function

The smooth ER undertakes diverse metabolic processes including lipid synthesis, carbohydrate metabolism, detoxication, and Ca^2+^ storage, while rough ER is involved in the synthesis of secretory and membrane proteins as well as co- and post-translational modifications such as protein folding, glycosylation, secretion, and degradation.^2^ Protein folding occurs with a high error rate and many factors, such as nutrient deprivation, modified glycosylation, depletion of Ca^2+^, oxidative stress, DNA damage, and imbalance in energy levels, can lead to an accumulation of unfolded and misfolded proteins causing ER stress.^31^ To alleviate ER stress, mammalian cells develop three main adaptive and interwire mechanisms including ER-associated degradation (ERAD), unfolded protein response (UPR), and reticulophagy.^32^

The ERAD targets proteins with folding difficulties, misfolded domains, or those lacking specific protein partners, returning them to the cytosol for polyubiquitylation and subsequent degradation by the 26S proteasome.^33^ Under a normal state, the molecular chaperone glucose-regulated protein 78 (GRP78) forms a complex with three signaling pathways including inositol requiring enzyme 1α (IRE1α), protein kinase R like endoplasmic reticulum kinase (PERK), and activating transcription factor 6 (ATF6) and drives the UPR in an inactivated form.^31^ However, in the presence of ER stress or the accumulation of unfolded proteins, GRP78 dissociates from the complex, resulting in a translocation of ATF6 to the Golgi to be activated and releasing an NH_2_-domain fragment into the nucleus to regulate specific target genes to rescue protein homeostasis. If it fails, the UPR is directed toward initiating programmed cell death.^34,35^

Finally, reticulophagy is a clearance process of excess ER membranes and proteins to reduce ER stress.^36,37^ The process involves the binding of lipidated LC3 on autophagic membranes to the translocon component, SEC62 preprotein translocation factor, to form an ER-derived vesicle which contains molecular chaperones and folding factors but not the degradation factors.^36^ As a consequence, the formed complex triggers the engulfing process by endolysosomes via the endosomal sorting complex required for transport (ESCRT)-III components including charged multivesicular body protein 4B (CHMP4B) and vacuolar protein sorting-associated protein 4A (VPS4A).^37^ Many studies have clarified both beneficial and deleterious correlations between ER stress and CVDs.^32^ For example, tunicamycin-induced ER stress in mice via inhibition of N-linked glycosylation of ER proteins showed an enlarged cytosol, formation of mitochondrial clusters, increased amount of the rough ER network as well as alteration of mitochondrial function and biogenesis with heterogeneous shape and size and fragmented cristae during ER stress.^38^

Recent discoveries have revealed that mitofusin 2 (MFN2) functions as a crucial tether between ER and mitochondria, maintaining the stability and functional lifespan of mitochondrial-associated ER membranes (MAMs) and mitigating the UPR by boosting ER ATP supply through mitochondria during ER stress.^39^ It was reported that splicing of MFN2 results in two variants, ER mitofusin 2 tethers (ERMIT2) which are localized at the “bridge” and facilitate the connection between the ER and mitochondria by interacting with other mitofusins and the ER mitofusin 2 (ERMIN2) variant which regulates ER morphology.^40^

## Impacts of Mitochondrial Irregularities and ER Stress in the Pathogenesis of Heart Failure (HF)

### Mitochondrial Dysfunction and HF

HF results from either functional or structural impairment of myocardium, causing an inadequate cardiac output and failing to keep up with the metabolic needs of the body. Systolic HF, also known as HF with reduced ejection fraction (HFrEF), and diastolic HF, termed HF with preserved ejection fraction (HFpEF), are two common categories differentiated by left ventricular (LV) ejection fraction, each with distinct etiology, pathophysiology, and treatment strategies.^41^ An adult human heart is estimated to consume 6 kg of ATP per day to pump blood with 95% of ATP derived from mitochondria and 5% from glycolysis and the citric acid cycle (TCA or Krebs cycle) for contraction and various ion pumps.^26^ In HF patients, cardiac ATP flux through creatine kinase (known as primary energy storage of the heart) was reduced by 50% in mild-to-moderate HF.^42^

Heart contraction is determined by intrinsic factors via Ca^2+^ and ATP and extrinsic hemodynamic factors, such as the elasticity and contractile state of arteries and veins, alongside the blood’s volume and viscosity with decreased contractile function being a typical characteristic of HFrEF.^43^ Cardiac contraction requires an increase in intracellular Ca^2+^ levels from approximately 150 nM at rest state to 600 nM to achieve 50% maximal activation.^44^ During systole, a small amount of extracellular Ca^2+^ enters the cytosol via the voltage-gated L-type Ca^2+^ channel and binds to the ryanodine receptor 2 (RyR2) to trigger more Ca^2+^ release from the sarcoplasmic reticulum (SR) into the cytosol.^44,45^ As a result, intracellular Ca^2+^ levels increase approximately 10-fold, allowing Ca^2+^ to bind to the myofilament protein troponin C and initiate contraction. In the next stage, muscle relaxation begins with the removal of Ca^2+^ from the cytosol followed by the dissociation of Ca^2+^ from troponin. Especially, the Na^+^/Ca^2+^ exchanger (NCX) and plasma membrane Ca^2+^-ATPase pump out approximately 30% of Ca^2+^ and the rest is returned to SR via the cardiac SR Ca^2+^-ATPase (SERCA2a).^45^

Mitochondria rely on the mitochondrial Ca^2+^ uniporter (MCU) for Ca^2+^ uptake, while its extrusion is regulated by the Na^+^/Ca^2+^/Li^+^ exchanger (NCLX), allowing them to respond to changes in cytosolic Ca^2+^ and maintain intracellular Ca^2+^ homeostasis.^25,46^ An abnormal leak of Ca^2+^ from SR through RyR2 leads to mitochondrial Ca^2+^ overload which impairs mitochondrial functions, contributes to cytosolic Ca^2+^ overload, and ultimately plays a role in HF.^47^ Increased intracellular Ca^2+^ levels also activate calpain, a family of Ca^2+^-dependent neutral cysteine proteases observed in diseased hearts, and are implicated in cardiac cell death, hypertrophy, fibrosis, and inflammation.^48^ However, if mitochondrial Ca^2+^ is below the threshold level, it impairs the TCA cycle and ATP production while increasing nicotinamide adenine dinucleotide phosphate (NADPH) oxidation.^49^ Thus, maintaining Ca^2+^ homeostasis is critical for normal cardiac function.

Moreover, mitochondria are the main source of intracellular ROS, byproducts of normal cellular metabolism that can be produced from both endogenous and exogenous sources, in addition to peroxisomes and ER.^50^ An imbalance in ROS production and the antioxidant system contributes to CVDs.^51^ There are possible sources of ROS that have been proposed to the association with HF including the mitochondrial electron transport chain (ETC), NADPH oxidases, monoamine oxidases, xanthine oxidase, or uncoupled nitric oxide synthase.^52^ The guinea pig model has been extensively employed to demonstrate the important human HF features including prolonged Q-wave/T-wave (QT) interval and spontaneous arrhythmic sudden cardiac death, showing the significantly elevated mitochondrial ROS levels of myocytes in both resting and contracting LV of failing hearts.^51^ Besides, measuring serum derivative of reactive oxygen metabolites (DROM), a marker of ROS, in 201 patients with HFrEF showed a significantly increased level of DROM and positively correlated with other markers such as B-type natriuretic peptide (*r* = 0.34, *p* < 0.001) and high-sensitivity C-reactive protein (*r* = 0.57, *p* < 0.001).^53^ A study on monkey embryos and rat embryos illustrated the differentiation of mitochondria throughout the heart’s development.^54^ In both species, it was observed that during the early embryonic stages, mitochondria have few lamellar cristae and exhibit low oxidative phosphorylation activity, relying primarily on anaerobic glycolysis for ATP production.^54^ As maturation progresses, mitochondrial morphology undergoes significant changes, with cristae becoming more lamellar, thereby increasing the inner surface area of mature mitochondria and enhancing their function. In addition, the diameter of heart mitochondria was about twice that found in other organs.^54^

To maintain cellular fitness, mitochondria quantity and quality must be controlled through mitochondrial fusion, fission, and mitophagy processes.^25^ Peroxisome proliferator-activated receptor gamma coactivator 1-α (PGC-1α) is responsible for mitochondrial biogenesis and regulates mitochondrial fission and fusion.^26^ The fission process is mainly controlled by dynamin-related protein 1 (DRP1), fission protein 1 (FIS1), mitochondrial fission factor (MFF), and mitochondrial dynamics proteins of 49 and 51 kDa (MID49/51). In addition, hexokinase 1 (HK1), an enzyme involved in glycolysis, has been reported to regulate mitochondrial fission during starvation by forming ring-like structures that inhibit DRP1 binding to MFF or FIS1.^55^ Generally, mitochondrial fusion is mediated by outer mitochondrial membrane proteins, mitofusin 1/2 (MFN1/2), and inner mitochondrial membrane, protein optic atrophy 1 (OPA1).^26^ In cardiac DRP1 and MFN knockout mice, the LV was enlarged and ejection performance declined significantly.^56^ In HFpEF patients, the level of expression of glycolytic metabolites, including hexokinases, was significantly reduced. In particular, both HK1 and HK2, which convert glucose to glucose-6-phosphate in the first irreversible step of glycolysis, were reduced by 23% and 52%, respectively.^57^

Mitophagy is a vital process to selectively remove damaged mitochondria.^58^ In a transverse aortic constriction (TAC) mouse model, mitochondrial autophagy was activated from day 3 to day 7 and DRP1 was translocated and reached its peak on day 3 after TAC.^59^ Autophagy involves the formation of the autophagosomal membrane (Beclin-1), membrane elongation factors (autophagy-related proteins [ATG5-ATG12] and microtubule-associated protein 1 light chain 3 [MAP1-LC3]), and the cleavage of LC3-phosphatidylethanolamine conjugate (LC3–II) which localizes to the autophagosome membrane and subsequently fuses with a lysosome to form autophagosomes that degrade cell content.^60^ In the heart tissues of patients with idiopathic dilated cardiomyopathy, both the transcript and protein levels of autophagy markers and mediators were decreased.^61^

### ER Stress and HF

The ER is vital for numerous cellular functions, and multiple protective mechanisms are involved in maintaining ER homeostasis in cardiomyocytes.^32,34^ However, prolonged ER stress is associated with the pathology of cardiac hypertrophy and HF.^62−64^ A study reported that a TAC murine model developed cardiac hypertrophy after 1 week of prolonged ER stress with cardiac enlargement and an increase in LV wall thickness.^65^ The study also showed elevated mRNA levels of ER chaperones, including GRP78 calreticulin and atrial natriuretic peptide, in hypertrophic and failing hearts. Besides, ATF6 is a major mediator of UPR and maintains proteostasis and proteome integrity during cardiac growth.^31^ During cardiac hypertrophy, ATF6 and its targeted gene ras homologue enriched in brain (RHEB), an activator of the mammalian target of rapamycin complex 1 (mTORC1) pathway, were upregulated in mice.^66^ A study on heart samples from patients with end-stage HF and mitral valve replacement showed an elevated expression level of ER stress markers including GRP78, PERK, eukaryotic initiation factor 2α (eIF2α), and C/EBP homologous protein (CHOP) and its associated apoptosis markers compared with normal hearts.^67^ In human heart with dilated and ischemic cardiomyopathies, the structural proteins (Reticulon 1 and Reticulon 4) that stabilize the curvature of ER tubules and are involved in protein biosynthesis and cellular component transport along microtubules, ribosome-binding protein 1 (RRBP1) and kinectin, were upregulated which may alter ER structure.^63^ These results provided evidence of ER structure and shape alterations during ER stress events which are important in the pathogenesis of HF.

## Influences of Mitochondrial Dysfunction and ER Stress on Ischemic Heart Disease (IHD)

### Mitochondrial Dysfunction and IHD

Ischemic heart disease (IHD) or coronary artery disease (CAD) is characterized by the buildup of plaque in the blood vessels, leading to impairment in blood flow and oxygen supply to the myocardium and can ultimately lead to myocardial infarction (MI).^68^ The myocyte ATP content in patients with chronic CAD was considerably reduced after ischemia.^69^ The ROS levels during I/R injury could be from different sources such as increased xanthine oxidase formation, neutrophil respiratory burst, and damage of the mitochondrial ETC.^70^ In myocardial I/R mice deficient in various NADPH oxidase (NOX) isoforms, a significant decrease in myocardial infarct size was observed in mice lacking NOX1, NOX2, and NOX1/NOX2 but not in NOX4-deficient mice compared to wild-type mice.^71^ The study also observed upregulated levels of phosphorylated proteins in NOX1-deficient mice (e.g., protein kinase B [Akt] and extracellular signal-regulated kinase [ERK]) and NOX2-deficient mice (e.g., signal transducer and activator of transcription 3 [STAT3] and ERK), suggesting the interesting target pathways to cope with reperfusion damage associated with CAD.^71^ Remarkably, the failing hearts in both humans and rats showed a significant decrease in OPA1 levels, resulting in an increase in apoptosis and mitochondrial fragmentation, indicating that OPA1 plays a central role in I/R injury.^72^ Overexpression of OPA1 protected cardiomyocytes against I/R injury.^73^ In a mouse model with *OPA1* c.2708_2711delTTAG mutation, the infarct size of the mutant was greater than wild-type mice in both *in vivo* and *ex vivo* experiments.^74^ This study also demonstrated an impairment of mitochondrial Ca^2+^ uptake alongside an elevated late phase of repolarization, triggering a higher incidence of arrhythmia in mutant mice.^74^ Using bioinformatics analysis on two Gene Expression Omnibus (GEO) data sets (GSE62646 and GSE59867) and three distinct machine learning algorithms, three mitophagy-related genes closely correlated with MI, including ATG5, TOMM20, and MFN2, were identified.^75^

### ER Stress and IHD

Glucose is metabolized through multiple pathways including glycolysis, glycogen synthesis, the polyol pathway, the pentose phosphate pathway, and the hexosamine biosynthetic pathway (HBP).^76,77^ Of these, the HBP utilizes approximately 2–5% of the total glucose^77^ and produces a crucial metabolite for O-linked N-acetylglucosamine modification, a reversible post-translational modification that adds to serine and threonine residues of nuclear and cytoplasmic proteins, impacting various cellular functions.^78^ The role of HBP in cardiac physiology and pathophysiology is complex and its improper regulation has been linked to heart disease.^77,79^ The spliced X-box binding protein 1 (XBP1) is a downstream transcription factor in the IRE1 pathway that enhances gene expression of ER chaperones and molecules involved in ERAD.^32^ It also acts as an upstream activator of the HBP by directly targeting glutamine/fructose-6-phosphate amidotransferase-1 (GFAT1), a rate-limiting enzyme in HBP, as demonstrated in an engineered murine model.^80^

Tamoxifen is a frontline therapy used in breast cancer treatments that directly targets estrogen receptor-positive cancer cells by antagonizing estrogen receptors.^81^ Additionally, tamoxifen can trigger apoptosis via nonestrogen receptor-dependent mechanisms by suppressing mitochondrial respiration, stimulating mitochondrial lipid peroxidation, and inducing ER stress.^81,82^ ATF6, a branch of the UPR, demonstrated its protective role against ER stress in transgenic mice with cardiac-specific expression of a tamoxifen-activated form of ATF6.^64^ Upon tamoxifen treatment, ATF6 was activated in the hearts of transgenic mice, leading to an 8- and 15-fold increase in ER stress-inducible mRNA and protein expressions of GRP78 and GRP94.^64^ Notably, ATF6 can have both protective and harmful effects. Initially, ATF6 responses aim to protect the cell and resolve ER stress. However, if ER stress persists, the activated ATF6 pathways may enhance apoptosis to remove irreversibly damaged cells.^32,38,62^

## Involvement of Mitochondrial Abnormalities and ER Stress in Arrhythmia

### Mitochondrial Dysfunction and Arrhythmia

Arrhythmia is a broad spectrum of heart rate disorders and rhythm abnormalities caused by the disruption in the orderly electrical cycle of excitation and recovery through the myocardium.^83^ Mitochondria are imperative in the cardiac homeostatic regulation of intracellular cations such as Ca^2+^, Na^+^, and K^+^ that are involved in cardiac contractility, energetics, and electrical activity.^83^ Chloride ions (Cl^–^) balance major cations such as Na^+^, K^+^, and Ca^2+^, while also regulating cellular components such as lysosomes, mitochondria, endosomes, phagosomes, nucleus, and ER.^84^ Growing evidence has established that the Cl^–^ channel dysfunction has been associated with several CVDs including hypertension, IHD, myocardial hypertrophy, and HF.^84,85^ Hypochloremia is one of the electrolyte abnormalities found in HF patients and is associated with adverse outcomes.^86^*Ex vivo* analysis of atrial specimens from patients with atrial tachyarrhythmia showed not only an increase in pale and enlarged mitochondria with partial cristaeolysis and completely disrupted mitochondria but also reduced endogenous respiration compared to nonpaced controls.^87^ Similarly, in rabbits induced to develop tachycardiomyopathy, it was observed that the mitochondria swelled and shifted their localization to the intercalated discs.^88^ In addition, downregulation in the expression of genes related to oxidative phosphorylation, the TCA cycle, and fatty acid oxidation was detected in myocardium from patients with tachycardia and ventricular tissue from dogs with tachypacing-induced HF.^89^

ROS can affect all major ionic currents in the heart such as increasing the late Na^+^ current, L-type Ca^2+^ current, and NCX activity and causing a Ca^2+^ leak from the SR. Additionally, ROS can decrease peak Na^+^ current and Ca^2+^ uptake via SERCA, contributing to arrhythmia.^90^ In cardiomyocytes isolated from HF rabbits, the inhibition of late Na^+^ current, Ca^2+^/calmodulin-stimulated protein kinase II (CaMKII), and Ca^2+^ leak through RyR2 reduced both action potential duration prolongation and the incidence of delayed afterdepolarizations, while a mitochondrial ROS antioxidant mitigated the proarrhythmic effects triggered by RyR leak and elevated Na^+^ current.^91^ Likewise, enhanced RyR2 activity in ventricular myocytes from rats and catecholaminergic polymorphic ventricular tachycardia mice under β-adrenergic stimulation led to increased mitochondrial ROS production and Ca^+^ imbalance as well as exacerbation of Ca^2+^ extrusion from ER and mitochondrial depolarization which promoted proarrhythmic conditions. Nonetheless, these effects were alleviated by mitochondrial ROS scavenging.^92^ In addition, lysosomal Ca^2+^ plays an important role in ventricular myocyte contraction and correlates with arrhythmic risk in human-induced pluripotent stem cell-derived cardiomyocytes (hiPSC-CMs).^93^ However, it remains unclear whether lysosomal Ca^2+^ release directly influences membrane currents or affects Ca^2+^ levels in the SR or mitochondria of cardiomyocytes.

### ER Stress and Arrhythmia

The cardiac action potential is the key to a regular heart rhythm and is regulated via well-known channels including Na^+^, K^+^, Ca^2+^, and transporters.^45^ UPR is reported to contribute to cardiac arrhythmia through its regulation of multiple cardiac ion channels.^32^ Tunicamycin-induced activation of the UPR in hiPSC-CMs showed downregulation of currents across all major ion channels including Na_v_1.5, Ca_v_1.2, K_v_4.3, K_v_LQT1, and the human ether-a-go-go-related gene (hERG). These current reductions resulted from a distinct pattern of downregulation in which the PERK branch specifically targeted K_v_4.3, while the IRE1 branch downregulated the Ca_v_1.2 channel.^94^

## SGLT and SGLT2 Inhibitors

### SGLT Isoforms

Human solute carrier 5 (SLC5) family includes 12 different members expressed in tissues from epithelia to the central nervous system and is involved in the transportation of sugars, vitamins, amino acids, or smaller organic ions.^95^ SGLTs are one of the SLC5 subfamilies comprising seven members with different physiological functions, in which SGLT1 and SGLT2 are the most abundant isoforms.^8,96^ SGLT1 (encoded by *SLC5A1*) is significantly expressed in the small intestine, colon, trachea, kidney, and heart, whereas SGLT2 (*SLC5A2*) is predominantly distributed in the kidney and is not expressed in the heart.^8,9^ In the proximal tubule, SGLT2 is located in the first segment (S1) where it reabsorbs 90–97% of glucose, while SGLT1 in the S2 and S3 segments absorbs the remaining 3–10% of filtered glucose. SGLT3 (*SLC5A4*), acting as a glucose sensor, is located in the small intestine, while SGLT4 (*SLC5A9*) is found in both the small intestine and kidney and SGLT5 (*SLC5A10*) is present in the kidney.^8,95^ SMIT1 and SGLT6 (also called SMIT2) detected in the spinal cord, kidney, and brain are encoded by *SLC5A3* and *SLC5A11*, respectively.^95,97^

### SGLT Expression and Function in the Heart

#### SGLT1 (Encoded by *SLC5A1*)

In human tissues, two transcript variants of *SLC5A1* have been identified, including variant A consisting of 664 amino acids and variant B of 537 amino acids, with the glucose binding domain expected to consist of amino acid residues 457–460.^98^ SGLT1 has a high affinity for glucose at the late proximal tubule and early proximal tubule (*K*_m_ 0.5–2 mM) and transports Na^+^ and glucose with a 2:1 stoichiometry.^8,98^ SGLT1 is the major isoform expressed in the heart, particularly in the capillaries of the human heart rather than in myocyte sarcolemma.^99^ The expression of SGLT1 in the human heart was higher than that found in the kidney by approximately 10-fold.^100^ Conversely, the physiological function of SGLT1 in the heart remains unclear. Interestingly, the expression of SGLT1 is linked to various pathological processes in the heart including oxidative stress, inflammation, fibrosis, apoptosis, and mitochondrial dysfunction.^101^

In heart diseases, cardiac SGLT1 expression could be changed by the alterations in the utilization of energy sources.^101^ Cardiac SGLT1 mRNA expression was increased up to 7-fold in the failing heart tissues of patients and mice compared to healthy controls.^102^ Besides, SGLT1 expression was suppressed in a model of T1DM but increased to approximately 2- to 3-fold in patients with end-stage cardiomyopathy secondary to T2DM and T2DM mice.^102^ In addition, transgenic overexpression of SGLT1 in mice caused cardiac hypertrophy and LV dysfunction, while knockout of SGLT1 in protein kinase AMP-activated noncatalytic subunit gamma 2 (PRKAG2) cardiomyopathy reversed the disease phenotype, implicating SGLT1 in the pathogenesis.^103^ On the other hand, the human heart expressed SGLT1 variant B, whereas variant A could not be detected and amplified in the heart. The truncated SGLT1 protein encoded by variant B lacked three transmembrane domains and residues involved in glucose and Na^+^ binding; therefore, cardiac SGLT1 did not contribute to overall glucose uptake.^98^ Overall, the role of SGLT1 in cardiac function remains uncertain.

#### SMIT1 (Encoded by *SLC5A3*)

Another member of the SGLT family, SMIT1, which has been identified in the human heart, shows mRNA expression approximately 10-fold lower than in the brain, while the mRNA level of SGLT1 is higher.^97^ It is reported that elevated levels of SMIT1 aggravated glucotoxicity effects on cardiomyocytes and increased their sensitivity whereas its deletion prevented the activation of NOX2 induced by high glucose condition.^97^ However, the physiological role of SMIT1 in the heart remains to be elucidated. SMIT1 and SMIT2 are responsible for cotransporting myoinositol with Na^+^ but have a low affinity for glucose and barely affect glucose uptake in the heart in both normoglycemic and hyperglycemic conditions, proposing that the pathophysiology of diabetic cardiomyopathy probably depends on a signaling cascade activated by SMIT1 which could link to the ionic changes downstream of SMIT1.^95−97^

### Current SGLT2 Inhibitors

**Figure 1:**
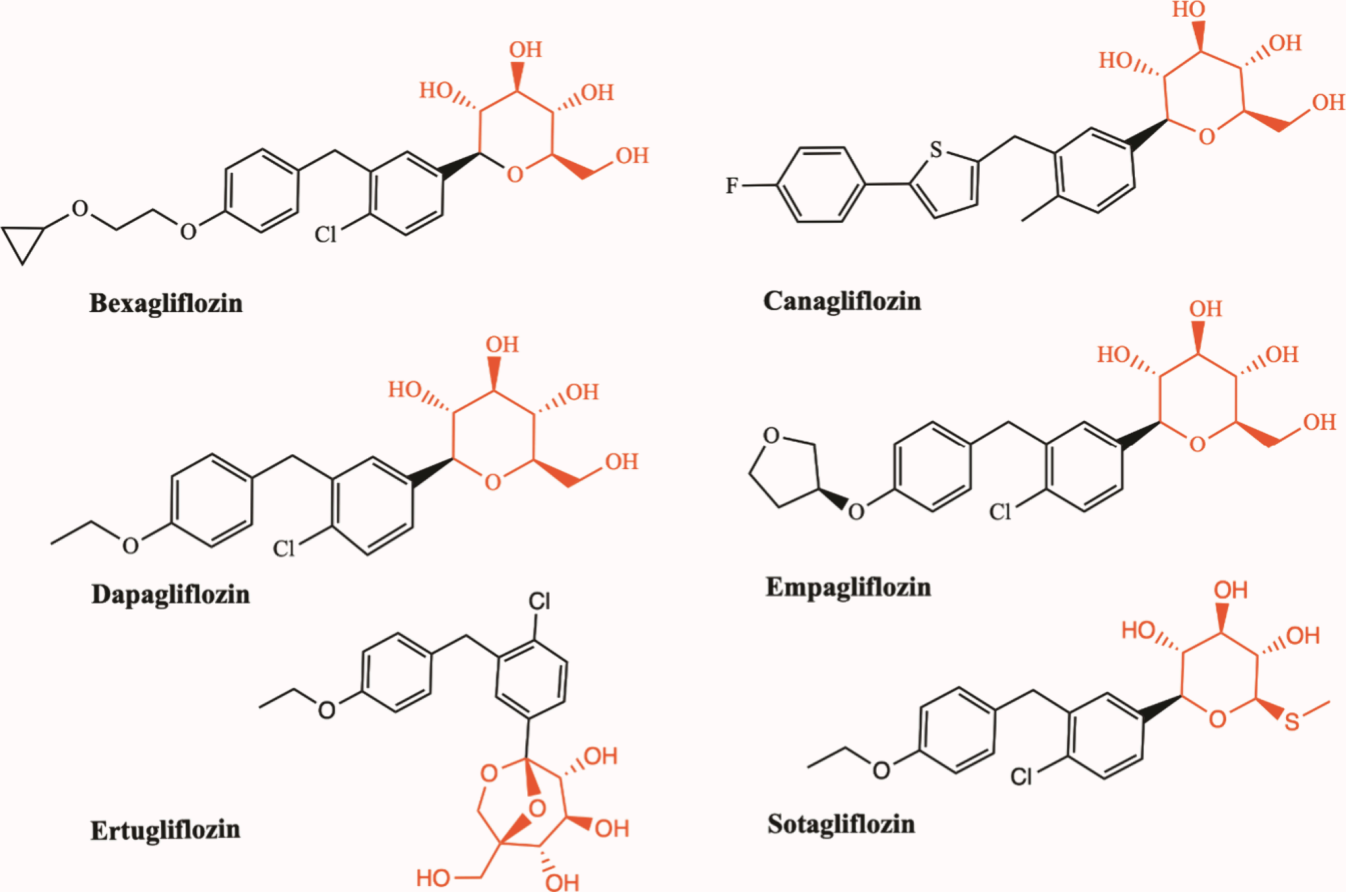
Structures of currently available SGLT2 inhibitors. All chemical structures, including bexagliflozin, canagliflozin, dapagliflozin, empagliflozin, ertugliflozin, and sotagliflozin, were derived from SMILES (PubChem). Sterically aligned functional groups are depicted in red. The figures were generated by ChemDraw version 20.0.0.38.

SGLT2 is responsible for the majority of the reabsorption of filtered glucose from tubular lumen.^8^ SGLT2 inhibitors are indicated for the treatment of T2DM to reduce the reabsorption of filtered glucose, thus promoting urinary glucose excretion.^104^ After the initial development of the first oral SGLT2 inhibitor (T-1095, a phlorizin prodrug) in 1999, various orally selective SGLT2 inhibitors were formulated.^105^ Nowadays, six SGLT2 inhibitors have been approved by the US FDA including canagliflozin (CANA) (Mar 2013), dapagliflozin (DAPA) (Jan 2014), empagliflozin (EMPA) (Aug 2014), ertugliflozin (ERTU) (Dec 2017), bexagliflozin (BEXA) (Jan 2023), and sotagliflozin (SOTA) (May 2023)^106−108^ (Figure 1) with established pharmacodynamics and pharmacokinetic profiles (Table 1). Recent landmark CV outcomes from two single large-scale trials (DAPA-HF and EMPEROR-Reduced) revealed unexpected CV mortality benefits with 14% reduction (HR 0.86, 0.76–0.98) from SGLT2 inhibition in patients with HFrEF regardless of age and sex and diabetic status, suggesting the unidentified cardioprotective mechanisms of SGLT2 inhibitors might result from the Na^+^ handling, energy homeostasis, and mitigation of cellular stress rather than be directly involved with glucose control.^16^

**Table 1:** Pharmacodynamics and Pharmacokinetics of SGLT2 Inhibitors^a^

Profiles	Canagliflozin (CANA)	Dapagliflozin (DAPA)	Empagliflozin (EMPA)	Ertugliflozin (ERTU)	Bexagliflozin (BEXA)	Sotagliflozin (SOTA)
Potency (SGLT2: SGLT1)	160: 1^109^	1,400: 1^110^	2,500: 1^111^	2,235: 1^111^	2,435: 1^112^	20: 1^113^
IC_50_ (nM) (hSGLT2: hSGLT1)	2.7: 710^114^	1.12: 1,391^115^	3.1: 8,300^114^	0.877: 1,960^116^	2: 5,600^112^	1.8: 36^117^
Urinary glucose excretion	77–119 g/day^118^	52–85 g/day^119^	3.1–90.8 g/day^120^	46.3–72.3 g/day^121^	19.1 L/h^107^	260–370 L/h^108^
Absorption (hours)	1–2^122^	0.5–1.3^123^	1.33–3.00^106^	0.5–1.5^106^	2–4^107^	1.25–4^108^
Distribution	PPB (%): 99	PPB (%): 91	PPB (%): 86	PPB (%): 93	PPB (%): 93	PPB (%): 93
	Vd (L): 83.5^106^	Vd (L): 118^106^	Vd (L): 85.5^106^	Vd (L): 74^106^	Vd (L): 262^107^	Vd (L): 9,000^108^
Metabolism	UGT1A9, UGT2B4^106^	UGT1A9^106^	UGT2B7, UGT1A3, UGT1A8, UGT1A9^106^	UGT1A9, UGT2B^106^	UGT1A9^107^	UGT1A9^108^
Excretion (%) (urine: feces)	33: 41^118^	75: 21^119^	54.4: 41.2^119^	50.2: 40.9^121^	40.5: 51.1^107^	57: 37^108^
Half-life (hours)	10.6–13.1^118^	12.9^119^	12.4^119^	16.6^121^	12^107^	21–35^108^
Main CV outcomes	CANVAS^124^ (NCT01032629, NCT01989754)	DECLARE-TIMI58^125^ (NCT01131676)	EMPA-REG^126^ (NCT01131676)	VERTIS CV^127^ (NCT01986881)	BEST^128^ (NCT02558296)	SCORED^129^ (NCT03315143)
	*N* = 10,142 (T2DM and high CV risks)	*N* = 17,160 (T2DM and atherosclerotic CV risks)	*N* = 7,020 (T2DM and high CV risks)	*N* = 8,246 (T2DM and atherosclerotic CV risks)	*N* = 1,700 (T2DM and high CV risks)	*N* = 10,584 (T2DM and high CV risks)
	↓33% HF hospitalization (HR, 0.67; 0.52–0.87)	↓27% HF hospitalization (HR 0.73; 0.61–0.88)	↓35% HF hospitalization (HR 0.65; 0.50–0.85)	↓HF hospitalization (HR 0.70; 0.54–0.90)	↓CV death/HF hospitalization (HR 0.7; 0.4–1.1)	↓26% CV death and hospitalization (HR 0.84; 0.72–0.99)
			↓38% CV death (HR 0.62; 0.49–0.77)	↓All-cause mortality (HR 0.93; 0.80–1.08)		
		No difference in all-cause mortality (HR 0.93; 0.82–1.04)	↓32% All-cause mortality (HR 0.68; 0.57–0.82)			SOLOIST-WHF^130^ (NCT03521934)
						*N* = 1,222 (T2DM and HF)
						↓33% CV death and hospitalization (HR 0.67; 0.52–0.85)
Adverse effects	Genital mycotic infections (female), UTI, and increased urination^131^	Genital mycotic infections (female), nasopharyngitis, and UTI^132^	Genital mycotic infections (female), and UTI^133^	Genital mycotic infections (female)^134^	Genital mycotic infections (female), UTI, and increased urination^135^	UTI, volume depletion, diarrhea, and hypoglycemia^108^

a Abbreviations: CV, cardiovascular; HF, heart failure; HR, hazard ratio; PPB, plasma protein binding; SGLT, sodium-glucose cotransporter; T2DM, type 2 diabetes mellitus; UGT, UDP-glucuronosyltransferase; UTI, urinary tract infections; V_d_, volume of distribution.

The latest approval of SOTA, a dual SGLT1/SGLT2 inhibitor, for various HF conditions, including in patients without diabetes, raises concerns about its potential benefits and risks, particularly due to the absence of trial data for this specific population.^117,136^ In a zebrafish model of diabetes and HFrEF, a study found no significant differences in cardioprotective effects between the selective SGLT2 inhibitor EMPA and the dual SGLT1/SGLT2 blocker SOTA, with SOTA being less effective at high concentrations compared to EMPA.^137^ SOTA demonstrated early effectiveness in decompensated HF, whereas DAPA had a more pronounced effect in patients with more severe HF symptoms.^138^

CANA has a mild inhibitory effect on SGLT1 and increases circulating glucagon-like peptide-1 (GLP-1) levels with effects lasting a few hours, whereas SOTA’s effect can be sustained for up to a day.^139^ It is proposed that stimulation of the GLP-1 receptor might modify plaque composition and improve plaque stability, potentially reducing the risk of CV events in genetically predisposed hyperlipidemic rabbits.^140^ However, this study did not find a correlation between circulating GLP-1 levels and the progression of coronary plaque. Thus, it is important to clarify whether an increase in GLP-1 levels is sufficient to achieve a clinically significant impact on glycemic control and potentially provide CV protection. Additionally, the ambiguous role of mutant cardiac SGLT1 in glucose uptake raises doubts about the clinical relevance of SOTA in dual blocking, especially in nondiabetic patients. Although the approval of SOTA offers additional treatment options for HF patients, the more comprehensive data supporting the use of DAPA and EMPA highlight the need for further evidence to define the role of SOTA in HF treatment.

## Molecular Mechanisms of SGLT2 Inhibitors on Mitochondrial Abnormalities in the Heart

**Figure 2:**
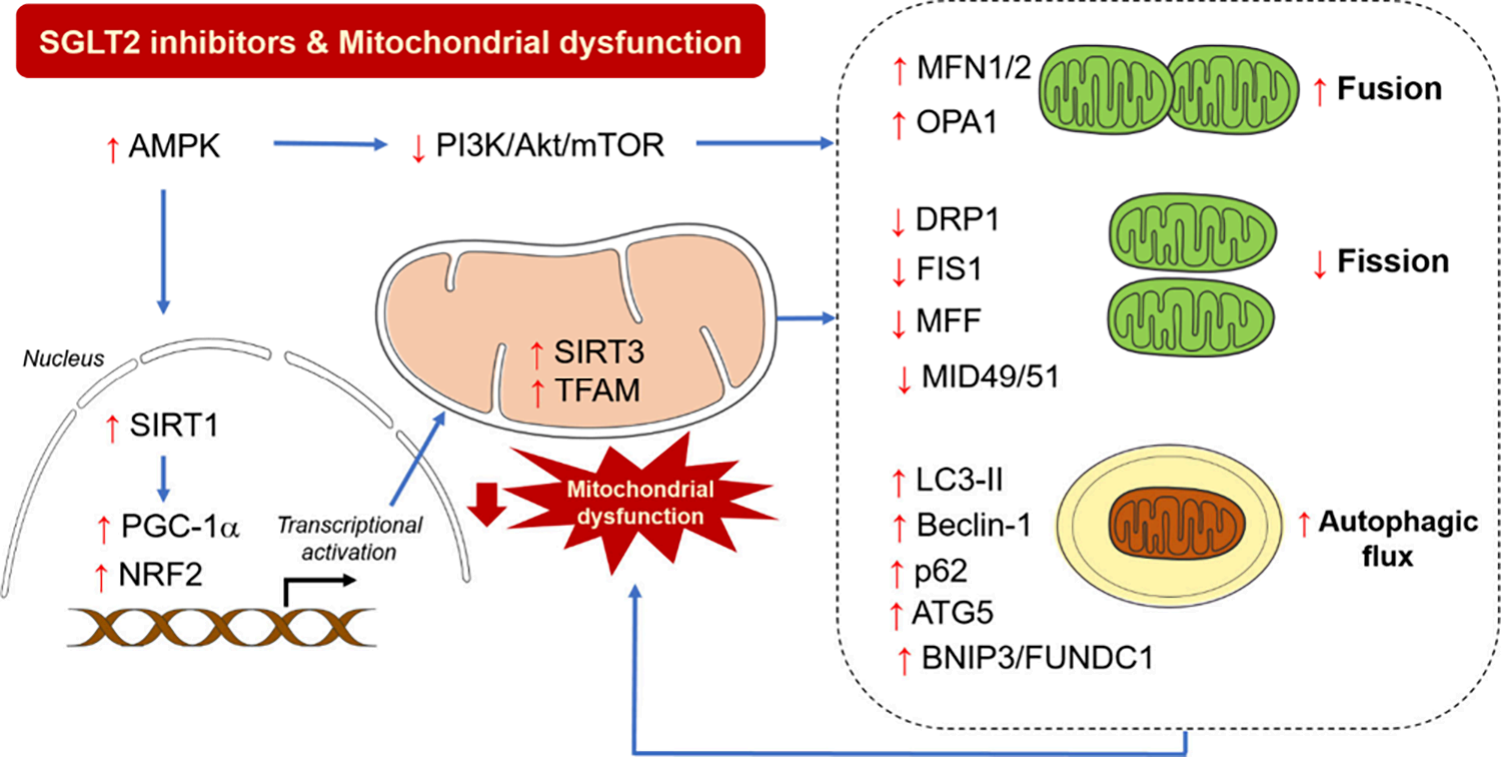
Potential impacts of SGLT2 inhibitors on mitochondrial dysfunction in heart diseases. The cardioprotective effects of SGLT2 inhibitors likely involve their beneficial impact on mitochondrial health and homeostasis. SGLT2 inhibitors enhance the upregulation of AMPK, SIRT1, PGC-1α, and NRF2 which are key regulators of crucial proteins for cellular balance; this activation boosts SIRT3 and TFAM, promoting mitochondrial fusion and autophagic flux while reducing fission. Additionally, AMPK activation reduces the PI3K/Akt/mTOR pathway, supporting mitochondrial health. The effects of SGLT2 inhibitors on mitochondrial markers are indicated by the red arrows (↑, increase; ↓, decrease). The illustrator was generated by Microsoft PowerPoint version 16.37. Abbreviations: Akt, serine/threonine protein kinase; AMPK, adenosine monophosphate-activated protein kinase; ATG5, autophagy-related protein 5; BNIP, BCL2 interacting protein; DRP1, dynamin-related protein 1; FIS1, fission protein 1; FUNDC, FUN14 domain-containing protein; LC3–II, LC3-phosphatidylethanolamine conjugate; MFF, mitochondrial fission factor; MFN1/2, mitofusin 1/2; MID49/51, mitochondrial dynamics proteins of 49 kDa and 51 kDa; mTOR, mammalian target of rapamycin; NRF2, nuclear factor erythroid-2 related factor 2; OPA1, optic atrophy 1; PGC-1α, peroxisome proliferator-activated receptor gamma coactivator 1-alpha; PI3K, phosphoinositide 3-kinase; SIRT1/3, sirtuin 1/3; TFAM, mitochondrial transcription factor A.

The remarkable CV benefits observed in SGLT2 clinical trials highlight the potential mechanisms that enhance mitochondrial health and function, thereby supporting ATP production in therapeutic strategies for CVDs.^16,141^ The pharmacological effects of SGLT2 inhibitors on the heart are outlined in Table 2 and their potential impacts on mitochondrial irregularities in heart diseases are illustrated in Figure 2.

**Table 2:** Pharmacological Effects of SGLT2 Inhibitors in the Heart^a^

Effects	Canagliflozin (CANA)	Dapagliflozin (DAPA)	Empagliflozin (EMPA)	Ertugliflozin (ERTU)	Bexagliflozin (BEXA)	Sotagliflozin (SOTA)
Oxygen demand	↑Peak VO_2_ (+2.4 mL/kg/min)^142^	↑Peak VO_2_ (+1.09 mL/kg/min)^143^	↑Peak VO_2_ (+1.21 mL/kg/min)^144^	NA	NA	NA
	↑Ventilatory anaerobic threshold (+1.5 mL/kg/min) and respiratory exchange ratio (+2.4 mL/kg/min)^142^		↑Peak O_2_ consumption^145^			
Na^+^/H^+^ exchange	↓mRNA levels of NHE-1 and NCX-1 in HFD-fed mice^146^	↓NHE-1 and heat shock protein 70 (Hsp70) in lipopolysaccharide-stimulated mouse cardio fibroblasts^148^	↓mRNA levels of both *SLC9C2* (NHE-11) and *SLC9A1* (NHE-1)^150^	↓Myocardial intracellular Na^+^ in the hearts of HFHS-fed mice^152^	NA	↓NHE-1 activity in a zebrafish model of diabetes and HFrEF^137^
	↓NHE activity (measured through low pH recovery after NH_4_^+^ pulse and reduced cytosolic Na^+^)^147^	↓NHE-1 levels in the heart of high-salt diet-fed Dahl salt-sensitive rats^149^	↓NHE-1 protein expression in angiotensin II-induced H9c2 cells^151^			
		↓NHE-1 expression in streptozotocin-induced diabetes rat^18^				
Fibrosis and hypertrophy	↓Body weight, fat mass, white adipose tissue weight, and adipocyte hypertrophy^153^	↓LAV index and LV mass index^155^	↓Hypertrophied cardiomyocyte area and LV thickness in HFD-fed mice^157^	↓Interstitial fibrosis and prevented diastolic dysfunction in the hearts of HFHS-fed mice^158^	NA	↓Cardiac hypertrophy and hypertrophic gene expression in a rat model^161^
	↓Collagen deposition, reversed weight increase for total heart and LV in isoprenaline-induced cardiac hypertrophy rats^154^	↓LV mass, systolic blood pressure, body weight, visceral and subcutaneous adipose tissue, insulin resistance, and high-sensitivity C-reactive protein in T2DM patients^156^	↓Angiotensin II-induced hypertrophy through inhibition of SGLT1 and NHE-1 expression^151^	↑LV function in a rat model of cardiac hypertrophy^159^		↓Cardiac hypertrophy and histological markers of cardiac fibrosis in transverse aortic constriction-induced mice^162^
	↓T2DM-induced basement membrane thickening and microvascular fibrosis^22^			↓Cardiac hypertrophy and remodeling via mTOR pathway^160^		

a Abbreviations: LAV, left atrial volume; LV, left ventricular; HFD, high-fat diet; HFHS, high-fat high-sucrose; HFrEF, heart failure with reduced ejection fraction; mTOR, mammalian target of rapamycin; NA, not available; NCX, Na^+^/Ca^2+^ exchanger; NHE, Na^+^/H^+^ exchanger; SGLT, sodium-glucose cotransporter; T2DM, type 2 diabetes mellitus; VO_2_, volume of oxygen consumption.

### Effect on ATP Production

Cardiomyocytes rely on a steady supply of ATP to maintain their proper function, a process that becomes disrupted in failing hearts.^3,26^ Studies have indicated that SGLT2 inhibitors enhance cardiac energy dynamics by boosting mitochondrial ATP production (Figure 2).^158,163−165^ After CANA treatment in a rat model of high-salt diet (HSD)-induced HFpEF, the ATP levels in the treated group showed an upward trend compared with the HSD-treated group.^166^ A detailed study found that SGLT1 was highly expressed, while SGLT2 was undetectable in human atrial tissues and isolated human cardiomyocytes.^165^ Moreover, CANA raised the intracellular ADP/ATP ratio in a hyperglycemic environment by inhibiting glucose uptake through SGLT1, thereby restoring the balance between ADP and ATP.^165^ Interestingly, CANA protective effects involved suppressing NADPH oxidase activity and reducing the nitric oxide synthase uncoupling via SGLT1/Rac1/adenosine monophosphate-activated protein kinase α2 (AMPKα2), indicating that SGLT2 inhibitors could protect cardiomyocytes through SGLT1.^165^ Exposure to DAPA rescued the cellular ATP depletion caused by palmitic acid in human umbilical vein endothelial cells by preserving mitochondrial biogenesis via the sirtuin 1 (SIRT1)/PGC-1α pathway.^164^ EMPA substantially increased ATP production and glucose oxidation in *db*/*db* mice hearts without changing the cardiac efficiency and oxygen consumption.^167^ Similarly, ERTU exposure preserved the maximal ATP production and improved cardiac energetics in the hearts of high-fat high-sucrose (HFHS) diet-induced diabetic cardiomyopathy mice.^152,158^

Furthermore, EMPA showed its pronounced impact by elevating both cytosolic and mitochondrial ATP levels in the ischemic reperfusion hearts from a mouse model through the inhibition of Na^+^/H^+^ exchanger-1 (NHE-1) and Na_v_1.5 channels, thereby recovering the damaged area and improving cardiac robustness.^163^ Real-time monitoring of ATP levels in a living state confirmed that EMPA rapidly restored mitochondrial ATP production to baseline levels within 3 h of a single dose. This immediate response to myocardial energy metabolism was sustained with these qualitative changes maintained throughout the entire cell over a period of 10 weeks.^163^ However, whether the mechanism underlying the effects of SGLT2 inhibitors involves direct NHE inhibition or off-target effects remains controversial.^168^ Treatment with EMPA failed to directly inhibit NHE-1 or reduce intracellular Na^+^ levels in isolated cardiomyocytes and Langendorff-perfused hearts from mice, rats, and guinea pigs, raising doubts about their beneficial effects on myocardial NHE-1 and Na^+^ handling in failing hearts.^169^

The effect of ERTU was observed not only on the pathological cardiac remodeling but also on the cardiac contractile reserve ability measured as the rate pressure product (RPP).^152^ Mice fed with HFHS showed impairments in contractile function and cardiac energetics; however, these impairments were restored to levels similar to or better than those of the control group by ERTU treatment.^152^ Based on gene ontology (GO) analysis using normalized enrichment scores, ERTU upregulated gene sets linked to cardiac oxidative phosphorylation and fatty acid metabolism with the top 20 gene sets involved in mitochondrial structure and function such as cellular respiration, oxidative phosphorylation, ETC, and fatty acid β-oxidation in both diabetic and nondiabetic mice.^158^

### Regulation of Mitochondrial Membrane Potential (MMP)

Mitochondria oxidize pyruvate (from glucose or lactate), fatty acids, and amino acids to produce energy through the Krebs cycle and redox reactions,^5^ creating a proton gradient across the mitochondrial inner membrane known as the mitochondrial membrane potential (MMP) which drives ATP synthesis as protons re-enter through complex V.^170^ MMP was recently described as being unevenly distributed across the inner mitochondrial membrane^171^ with its spatial regulation at the cristae junction controlled by mitochondrial Ca^2+^ uptake 1 (MICU1), acting as a relay for bioenergetics.^172,173^ The cristae junctions of mitochondria facilitate the exchange of ions and molecules between subdomains, regulating mitochondrial Ca^2+^ uptake, MMP, oxidative stress, and ATP production, with the stability of these processes being controlled by OPA1 and MICU1.^172,173^ Typically, silencing OPA1 and MICU1 destabilizes the cristae junctions, causing constriction of the inner mitochondrial membrane and subsequent mitochondrial fission with a notable shift in the MMP ratio toward the inner boundary membrane.^171^

Quantification analysis of MMP in palmitic acid-treated HL-1 mouse cardiomyocytes by flow cytometry showed that CANA increased MMP significantly.^174^ CANA also ameliorated the damage and restored mitochondrial function by improving MMP and ATP production in high glucose-induced H9c2 cardiomyoblasts.^175^ In H9c2 cardiomyoblasts treated with doxorubicin, DAPA was found to significantly increase MMP, indicating the improvement of mitochondrial functions by SGLT2 inhibitor.^176^ Conversely, LY294002, a specific phosphoinositide 3-kinase (PI3K) inhibitor, or cells transfected with nuclear factor erythroid-2 related factor 2 (NRF2) siRNA reversed the protective effect of DAPA, suggesting the activation of PI3K/Akt/NRF2 signaling by DAPA.^176^

In insulin-resistant metabolic rats, DAPA, but not insulin, substantially prevented MMP loss in isolated cardiomyocytes.^177^ EMPA and ERTU also protected mitochondrial MMP loss.^20,152^ In the cardiomyocytes isolated from 8-week-old mice with cardiorenal syndrome type-3 (CRS-3), a secondary cardiac dysfunction to acute kidney injury, EMPA treatment promoted an increase in mitochondrial ATP levels and stabilized the MMP in cardiomyocytes after the injury.^20,163^ In addition, treatment with ERTU in a mouse model of HFHS diet-induced diabetic cardiomyopathy also improved energetic deficit and contractile dysfunction as well as lowered intracellular Na^+^ levels in isolated cardiomyocytes, indicating the improvement of MMP.^152^

### Influence on ETC Complex Activity

The generation of ATP relies on the efficiency of the ETC within the inner mitochondrial membrane, where electrons undergo sequential transfer across protein complexes, culminating in ATP synthesis.^1^ In a rat model with myocardial I/R injury, complex II to V were no different across all groups treated with DAPA including those pretreated before ischemia, during ischemia, and at the onset of reperfusion.^178^ However, DAPA treatment elevated the expression of complex I in the ETC, indicating its role in attenuating the depletion of cardiac energy metabolism in response to I/R injury.^178^ Nonetheless, EMPA improved mitochondrial respiration via maintaining complex I and II activities in both human coronary artery endothelial cells and diabetic rats.^23,179^ Next-generation sequencing and the GO analysis platform found that 20 of 1,418 GO gene sets enriched by ERTU were correlated with oxidative phosphorylation and fatty acid metabolism and independent of diet in mice.^158^

### Impact on Mitochondrial Dynamics

Mitochondria are a dynamic structure, undergoing continuous change of shape through fusion and fission processes which are crucial for cellular functions.^25^ Any alterations in their morphology or internal matrix composition can disrupt mitochondrial functions, leading to cell dysfunction or death.^26^ In acute MI rats, DAPA treatment showed a trend of improvement in both mitochondrial morphology and structure within the infarcted myocardium.^180^ Moreover, the study revealed that DAPA raised the total number of mitochondria per cardiomyocyte and increased mitochondrial counts, indicating that the mitochondrial serine/threonine protein phosphatase family member 5 (PGAM5) and DRP1 pathway plays a role in conferring the associated benefits of DAPA.^180^ To maintain mitochondrial homeostasis, EMPA suppressed FIS1 upregulation, and activated BCL2 interacting protein 3 (BNIP3) expression, leading to the reduction in mitochondrial size and autophagic vacuole number after MI in diabetic rats.^21^ Besides, EMPA restored the irregular swelling and repaired the fractured and fuzzy cristae of mitochondria in cardiomyocytes of CRS-3 mice as well as preserved mitochondrial length and decreased the percentage of cardiomyocytes with rounded mitochondria.^20^

Moreover, heat shock protein 70 (Hsp70) serves as a crucial cellular chaperone involved in various protein folding processes, maintaining protein homeostasis and impacting human health.^181^ A study demonstrated that DAPA inhibited the NHE-1 and Hsp70 interaction in mouse cardiac fibroblasts through an AMPK phosphorylation-dependent manner, potentially attenuating the pro-inflammatory state associated with diabetes.^148^ However, the relationship between NHE-1 and Hsp70 in the context of SGLT2 inhibitor use remains unclear, necessitating further investigations to clarify their impacts on cardiomyocytes.

### Contribution to Autophagy and Apoptosis

Apoptosis is triggered by activated caspases to remove abnormal or unnecessary cells, while autophagy is a cytoprotective process where cytoplasmic contents are trapped within the autophagosome, fused with lysosomes, and degraded.^182^ To remove accumulated dysfunctional mitochondria, CANA augmented the protein expression of autophagic markers, LC3, p62, and AMPK, in a vitamin D_3_ plus nicotine-induced rat model of vascular calcification.^183^ Similarly, DAPA treatment restored autophagy flux by upregulated autophagy and autophagosome-related proteins including LC3B–II, ATG5, and Beclin-1 as well as autophagic flux marker p62 leading to the accumulation of autophagosomes in an I/R model.^184^ It is noted that DAPA acted via AMPK/mTOR/OPA1-mediated mitochondrial autophagy to attenuate myocardial injury.^185^

In addition, EMPA directly inhibited NHE-1 activity in cardiomyocytes to control excessive autophagy in response to starvation and effectively rescued cells from death aggravated by overexpressed NHE-1.^186^ In mouse cardiac microvascular endothelial cells (CMECs), EMPA increased biomarkers of autophagic flux (decreased p62 and increased LC3B–II), promoted mitochondria-lysosome interaction, and upregulated AMPK phosphorylation and unc-51-like kinase 1 (ULK1) expression.^187^ Furthermore, genetic silencing of AMPKα1 or FUN14 domain-containing protein 1 (FUNDC1) (a mitophagy receptor) abolished the beneficial effects of EMPA on both the myocardial microvasculature in a mouse model of myocardial I/R injury and CMECs, confirming the direct actions of EMPA in protecting cardiac microvascular I/R injury via AMPKα1/ULK1/FUNDC1/mitophagy pathway.^187^

### Modulation of ROS Production

Abnormal mitochondria are a primary generator of ROS, contributing to cellular damage.^170^ Treatment with CANA markedly reduced lipid peroxidation and ROS generation in H9c2 cardiomyoblasts induced by high glucose.^175^ CANA also lessened the expression of cardiac advanced oxidation protein products from LV myocardial tissue and NOX4 protein in Dahl salt-sensitive (DSS) rats.^166^ Furthermore, DAPA restored the antioxidant capacity system by activating the PI3K/Akt signaling and regulated NRF2 expression to improve mitochondrial function in H9c2 cells.^176^ In diabetic rats, complex I in the oxidative phosphorylation system was impaired, leading to increased mitochondrial ROS.^179^ However, EMPA treatment successfully restored complex I function most likely via AMPK-mediated pathways.^179^ Feeding male mice an HFHS diet led to an increase in the rate of hydrogen peroxide release from freshly isolated cardiac mitochondria and a decrease in the maximal rate of ATP production.^158^ Administration with ERTU reduced the hydrogen peroxide release and preserved the maximal ATP synthesis rate. Besides, histological staining showed an increase of 4-hydroxynonenal level, reflective of myocardial oxidative stress inhibited by ERTU treatment.^158^ Taken together, these results show that SGLT-2 inhibitors diminish both cellular and mitochondrial oxidative stress.

### Alteration in Cellular Ca^2+^ and Na^+^ Handling

In heart diseases, both intracellular Na^+^ and Ca^2+^ levels are increased, where Na^+^ overload disrupts both cellular and mitochondrial Ca^2+^ fluxes and impairs the balance between bioenergetic supply and demand.^3^ After short-term exposure to DAPA, the amplitude of shortening in ventricular myocytes of streptozotocin (STZ)-induced diabetic rats was substantially reduced; however, this effect diminished with long-term exposure to the drug, suggesting the development of tolerance.^188^ In addition, L-type Ca^2+^ current was suppressed in STZ-induced cardiomyocytes and further reduced by DAPA, suggesting its negative inotropic effects on the heart.^188^

Exposure to CANA, DAPA, and EMPA reduced cytosolic Na^+^ levels and impaired NHE-1 activity in mouse cardiomyocytes.^147^ In addition, a structural model study on the interaction of SGLT2 inhibitors to NHE showed that all three SGLT2 inhibitors possess a high binding affinity to the extracellular Na^+^ binding site of NHE and suggested that SGLT2 inhibitors exhibit an off-target effect on NHE-1.^147^ Additionally, treatment with EMPA normalized the levels of increased cytosolic Na^+^ and Ca^2+^ during diastolic and systolic states by inhibiting NHE flux in both rabbits and rats.^189^ Several studies have shown that SGLT2 inhibitors can effectively reverse late Na^+^ current as a putative mechanism as demonstrated in both human cardiomyocytes and murine models of HFrEF and HFpEF.^190−192^ In mice, EMPA was unable to block the reverse activity of the cardiac NCX isoform which is functionally coupled with NHE-1.^193^ However, it targeted the late Na^+^ current to lower abnormal Ca^2+^ levels in the heart by binding to Na_v_1.5 and inhibiting CAMKII phosphorylation which are important regulators of the late Na^+^ current.^192,193^

### Modification of Iron Homeostasis

Many patients with HF exhibit an iron-deficient condition which can restrict erythropoiesis in erythroid precursors and impede ATP production in cardiomyocytes.^194^ In the DAPA-HF trial, 43.7% of subjects presented with iron deficiency which was associated with worse outcomes than those observed in iron-replete individuals.^195^ An increase in hematocrit and significant reductions in transferrin saturation, ferritin, and hepcidin, along with an increase in total iron-binding capacity and soluble transferrin receptor were also observed in the patients who received DAPA.^195^ Collectively, this study suggested that DAPA increased iron utilization, likely due to enhanced erythropoiesis, and that the clinical benefits of DAPA were independent of baseline iron status. In line with these findings, studies have shown that erythropoietin levels increased in 54 obese patients treated with DAPA^196^ and in 44 diabetic patients on EMPA for 3 months,^197^ with similar effects observed in 90 patients with T2DM and CAD during a 6-month extension study.^198^

Aptamer-based proteomics analysis of 3,713 proteins in plasma samples from 72 participants mostly with T2DM revealed that EMPA significantly affected levels of 43 proteins, 6 related to cardiomyocyte, 5 iron handling proteins, and 1 in sphingolipid metabolism which plays a role in several pathological conditions including CVDs.^199,200^ In DSS rats treated with HSD to induce HFpEF, iron overloading, and lipid peroxidation, CANA could counteract these detrimental effects.^201^ Moreover, the Kyoto Encyclopedia of Genes and Genomes (KEGG) analysis and GO database for functional enrichment analysis revealed 9 out of the top 30 significant genes associated with ferroptosis, suggesting that ferroptosis might be a key mechanism involved in the protective effects of CANA in rats.^201^

## Role of SGLT2 Inhibitors on ER Stress in the Heart at the Molecular Level

**Figure 3:**
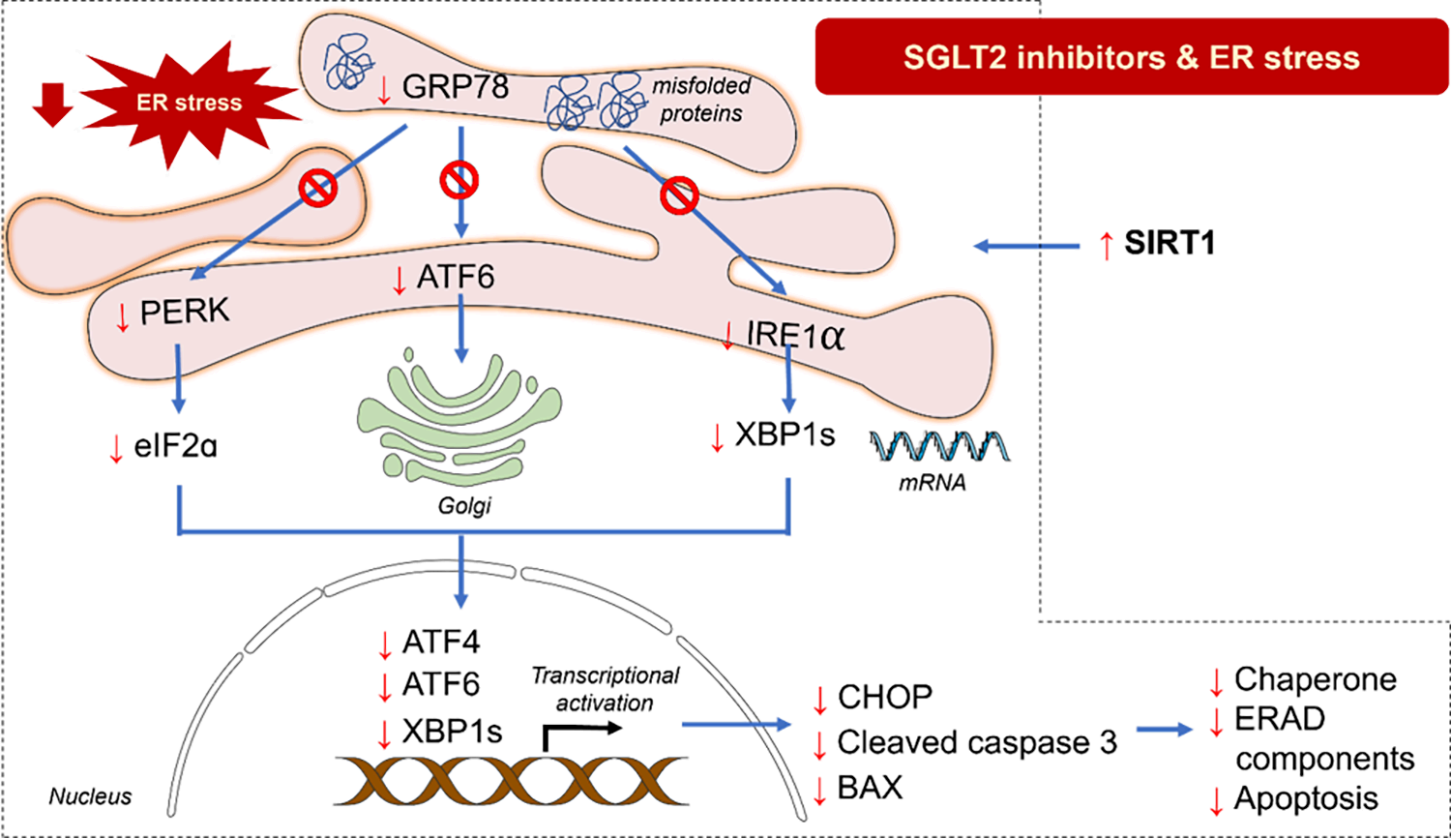
The influences of SGLT2 inhibitors on ER stress in heart diseases. SGLT2 inhibitors trigger the activation of SIRT1 which potentially inhibits GRP78 and three key transducers of the UPR including PERK, ATF6, and IRE1α. Consequently, stress-responsive transcription factors such as ATF4, ATF6, and spliced XBP1 are suppressed, resulting in decreased levels of chaperones, ERAD components, and apoptosis to relieve ER stress. The effects of SGLT2 inhibitors on ER markers are indicated by the red arrows (↑, increase; ↓, decrease). The illustrator was generated by Microsoft PowerPoint version 16.37. Abbreviations: ATF6, activating transcription factor 6; BAX, BCL2 associated X; CHOP, C/EBP homologous protein; eIF2α, eukaryotic initiation factor 2α; ER, endoplasmic reticulum; ERAD, endoplasmic reticulum-associated protein degradation; GRP78, glucose-regulated protein 78; IRE1α, inositol requiring enzyme 1α; PERK, protein kinase R like endoplasmic reticulum kinase; SIRT1, sirtuin 1; XBP1s, sliced X-box binding protein 1.

The involvement of ER stress in CVD is significant attributed to its contribution to inflammation and oxidative stress.^65^ Besides, ER stress induces apoptosis via UPR and causes negative effects in myocytes and endothelial cells.^32^ Therefore, targeting ER stress, particularly the UPR, emerges as a promising therapeutic approach for managing cardiac conditions. SGLT2 inhibitors have shown promising potential in alleviating ER stress, thereby protecting the heart, as summarized in Figure 3. Treatment with DAPA was reported to reduce all ER stress-associated proteins including GRP78, p-PERK, eIF2α, activating transcription factor 4 (ATF4), and CHOP in cardiac tissues of STZ-induced diabetic rats.^17^ In line with the above results, administration of DAPA in rats with LV dilatation and functional decline effectively suppressed the expression of ER stress-associated proteins (e.g., GRP78, CHOP, p-PERK/PERK ratio, eIF2α, and ATF4) in the LV of these rats compared to control.^202^

Another study also found that DAPA reduced ER stress and apoptosis in a rat model of mitral regurgitation-induced HF and injured cardiomyocytes mediated by angiotensin-II via inhibiting the eIF2α-ATF4-CHOP pathway.^203^ The protein expression levels of an ER stress biomarker GRP78 and compensatory UPR pathway (PERK/eIF2α/ATF4) as well as ER stress-related pro-apoptotic factors (e.g., CHOP and cleaved caspase 12) were also markedly decreased in DAPA treated rats with long-term β-adrenergic receptor activation with isoproterenol.^204^ In addition, high glucose treatment in H9c2 cells upregulated ER stress and apoptosis-associated proteins including GRP78, eIF2α, CHOP, ATF4, BCL2 associated X (BAX), and cleaved caspase 3.^18^ NHE-1, a ubiquitous membrane-bound enzyme, and protein kinase C β-II isoforms, key regulators in a variety of cell functions, were increased in cultured cardiomyocytes. However, treatment with DAPA rescued the expression of these proteins, suggesting that DAPA may act as a suppressor of apoptosis mediated by high glucose via mitigating the rise in levels of ER stress proteins.^18^

It is noteworthy that antihyperglycemic drugs including metformin, GLP-1 receptor agonists (e.g., exendin-4, liraglutide, albiglutide, and lixisenatide), and SGLT2 inhibitors (e.g., CANA, DAPA, and EMPA) reduced ER stress caused by tunicamycin or hyperglycemic conditions in cardiac endothelial cells isolated from coronary artery.^205^ This study also reported that these drugs suppressed the phosphorylation of IRE1α and PERK and reduced expression of the UPR regulator such as ATF6 and GRP78, suggesting that their cardioprotective properties could partially be attributed to their ability to reduce ER stress.^205^

In addition, EMPA blocked the PERK/ATF4/Beclin1 signaling thus suppressing ER stress-induced autophagy and alleviating myocardial I/R injury and cardiomyocyte apoptosis in mice and H9c2 cells.^19^ Treatment with EMPA also blocked mRNA transcription of ER stress-associated factors including ATF4, tumor necrosis factor receptor-associated factor 2 (TRAF2), and XBP1 in a dose-dependent manner in STZ-induced rats.^206^ Another member, ERTU demonstrated positive effects in mice with TAC-induced cardiac hypertrophy, notably increasing the phosphorylation of cardiac AMPK (Thr172) and AMPK-dependent Raptor (Ser792) phosphorylation.^160^ Additionally, this study observed ERTU reduced ER stress protection with a significantly less Akt-dependent tuberous sclerosis complex 2 (TSC2) (Ser939) phosphorylation and further limited different indicators of ER stress including ATF6, eIF2α, ATF4, and CHOP signaling thus reducing UPR target protein expression.^160^

## Conclusion and Future Directions

**Figure 4:**
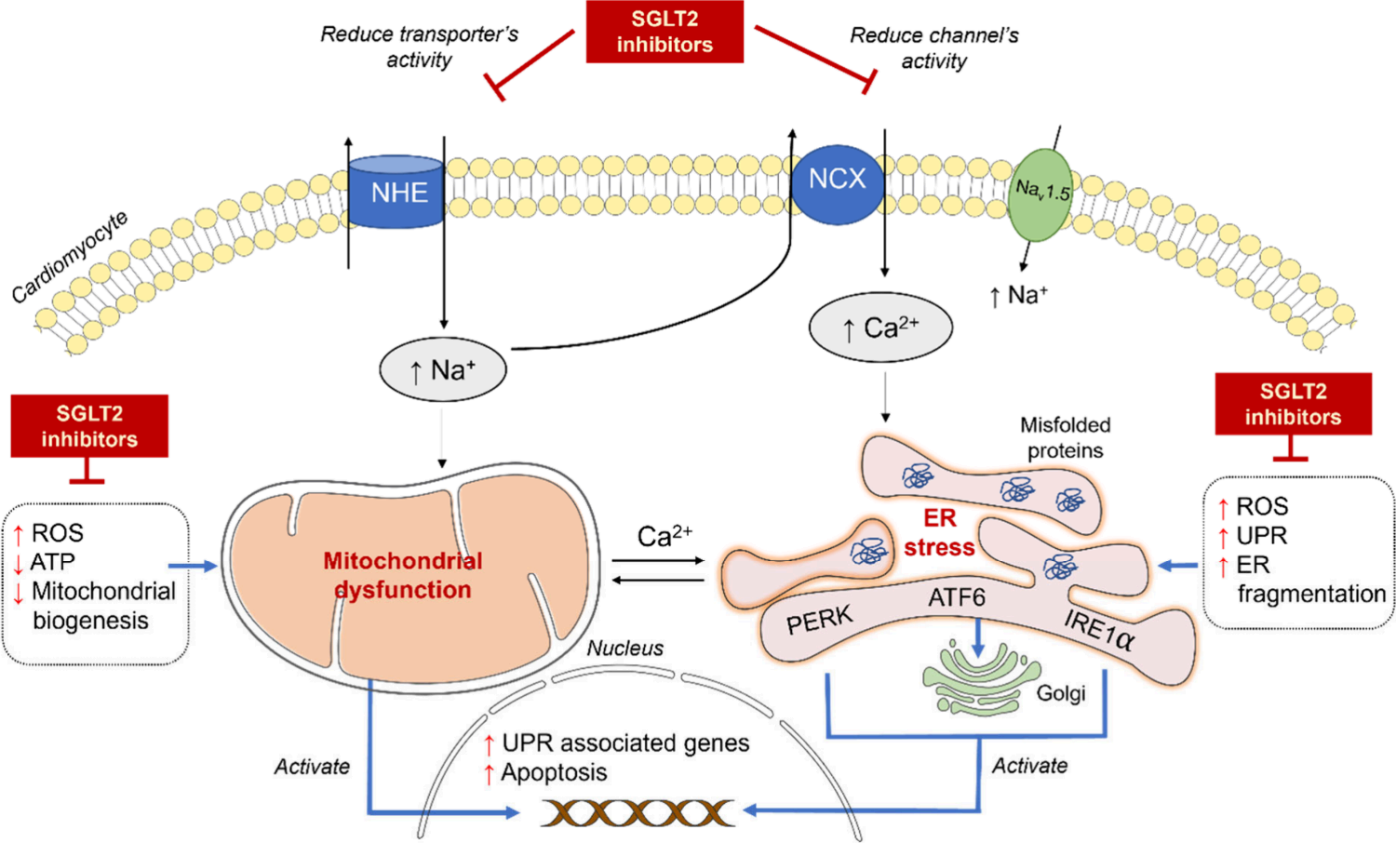
Overview of SGLT2 inhibitors’ potential effects on mitochondrial dysfunction and ER stress in cardiomyocytes. SGLT2 inhibitors affect the activity of NHE and NCX channels, reducing Na^+^ and Ca^2+^ overload in mitochondria and ER. Furthermore, these drugs regulate ROS levels and UPR, preserve the ER structure, and enhance energy production and mitochondrial biogenesis, ultimately safeguarding cardiomyocytes. The figure was generated by Microsoft PowerPoint version 16.37. Abbreviations: ATF6, activating transcription factor 6; ATP, adenosine triphosphate; ER, endoplasmic reticulum; IRE1α, inositol requiring enzyme 1α; NCX, Na^+^/Ca^2+^exchanger; NHE, Na^+^/H^+^ exchanger; PERK, protein kinase R like endoplasmic reticulum kinase; ROS, reactive oxygen species; UPR, unfolded protein response. Symbols: ↑, increase; ↓, decrease.

In summary, unraveling the complex cardiometabolic mechanisms of SGLT2 inhibitors requires comprehensive research and further studies on each inhibitor to elucidate their intricate roles in modulating ER stress and mitochondrial dysfunction in cardiomyocytes. In addition to their effects on NHE and NCX channels in Na^+^ and Ca^2+^ handlings, several lines of evidence have consistently reported that SGLT2 inhibitors restore mitochondrial biogenesis and alleviate ER stress by enhancing ATP production, preserving MMP, and targeting the UPR (Figure 4). Research indicates that SGLT2 inhibitors effectively downregulate the expression of NHE-1/NCX in the myocardium.^146−148,151,152^ Additional investigations should be conducted to elucidate and validate the mechanisms by which SGLT2 inhibitors influence the ETC complexes within the dysfunctional mitochondria in the heart. Furthermore, while SGLT2 inhibitors have been shown to regulate autophagic flux-associated proteins (e.g., p62 and LC3B–II), which facilitate the degradation of the inflammasome component nucleotide-binding domain, leucine-rich-containing family, and pyrin domain-containing-3 (NLRP3) during autophagy, the precise mechanisms of this process still require further investigation. Despite their potential to ease ER stress, several studies have focused on the PERK/ATF4/CHOP pathway and the direct molecular links of ER stress pathways; however, the protective effects of SGLT2 inhibitors on CVDs remain elusive.

All currently approved SGLT2 inhibitors, except BEXA and ERTU, are indicated for the treatment of HF; however, BEXA and ERTU have still demonstrated potential cardioprotective effects.^207^ Initially, the European Medicines Agency (EMA) temporarily approved DAPA and SOTA as add-on treatments to insulin for adults with T1DM and a body mass index above 27 kg/m^2^ under specialist supervision.^208,209^ However, the EMA later withdrew this recommendation due to potential adverse effects, particularly diabetic ketoacidosis.^210^ Concerns about the appropriateness of certain preclinical studies using STZ, which has been widely employed to develop models in diabetes research, have been raised. Either repeated low doses or a single high dose of STZ selectively damages pancreatic islet β-cells and mimics T1DM conditions, while coadministration of nicotinamide or prior exposure to a high-fat diet before administering a moderate dose of STZ is recommended for generating T2DM models.^211^ To ensure the reliability of such research, it is crucial to provide comprehensive methodological details and to interpret the results with caution.

Nevertheless, it remains uncertain whether all SGLT2 inhibitors share the same class of effects including their off-target cardioprotective abilities and the specific patient populations that benefit most. Some meta-analyses have compared the effects of SGLT2 inhibitors in specific populations, but debates persist. For example, while some clinical trials suggested that EMPA offers greater CV protection than CANA and DAPA in diabetic patients, others have not found significant differences between these drugs.^212−214^ Despite increasing evidence of the cardioprotective benefits of SGLT2 inhibitors, there is still limited data comparing these agents in nondiabetic patients and those with HFrEF or HFpEF. Therefore, further research in these specific populations is needed. Additionally, numerous preclinical studies have evaluated the effects of SGLT2 inhibitors across various disease models. However, substantial variations in dosage, administration routes for preventing or reversing pathological conditions, and treatment durations among these studies complicate the comparison of the results. Although often overlooked, reporting therapeutic plasma concentrations in patients and animal models could be essential for distinguishing clinically relevant effects from supratherapeutic off-target effects.

## Data Availability

All data generated or analyzed during the current study are included in this published article.

## Abbreviations:

Akt: serine/threonine protein kinase
AMPK: adenosine monophosphate-activated protein kinase
ATF: activating transcription factor
ATG5: autophagy-related protein 5
BNIP: BCL2 interacting protein
CHOP: C/EBP homologous protein
DRP1: dynamin-related protein 1
eIF2α: eukaryotic initiation factor 2α
ERAD: ER-associated degradation
ETC: electron transport chain
FIS1: fission protein 1
FUNDC: FUN14 domain-containing protein
GLUT: glucose transporter
GRP78: glucose-regulated protein 78
LC3–II: LC3-phosphatidylethanolamine conjugate
MFF: mitochondrial fission factor
MFN1/2: mitofusin 1/2
MID49/51: mitochondrial dynamics proteins of 49 kDa and 51 kDa
NADPH: nicotinamide adenine dinucleotide phosphate
NCLX: Na^+^/Ca^2+^/Li^+^ exchanger
NCX: Na^+^/Ca^2+^ exchanger
NRF2: nuclear factor erythroid-2 related factor 2
IRE1α: inositol requiring enzyme 1α
OPA1: optic atrophy 1
PERK: protein kinase R like endoplasmic reticulum kinase
PGC-1α: peroxisome proliferator-activated receptor gamma coactivator 1-α
PI3K: phosphoinositide 3-kinases
ROS: reactive oxygen species
SGLT: sodium-glucose cotransporter
SIRT1/3: sirtuin 1/3
TCA: citric acid cycle
TFAM: mitochondrial transcription factor A
UPR: unfolded protein response
